# Preventive Effect of Nuciferine on H_2_O_2_-Induced Fibroblast Senescence and Pro-Inflammatory Cytokine Gene Expression

**DOI:** 10.3390/molecules27238148

**Published:** 2022-11-23

**Authors:** Suphachai Charoensin, Wajaree Weera

**Affiliations:** 1 Division of Nutrition, School of Medical Sciences, University of Phayao, Phayao 56000, Thailand; 2 Unit of Excellence in Mathematical Biosciences, School of Medical Sciences, University of Phayao, Phayao 56000, Thailand; 3 Department of Mathematics, Faculty of Science, Khon Kaen University, Khon Kaen 40002, Thailand

**Keywords:** *Nelumbo nucifera*, nuciferine, senescence, inflammation, aging

## Abstract

Human dermal fibroblasts play an important role in skin homeostasis by producing and degrading extracellular matrix components. They have more replicative senescence when exposed to environmental and oxidative insults, resulting in human skin aging. However, this phenomenon can be mitigated by antioxidant phytochemicals. The aim of the present study was to investigate the potential of nuciferine (an alkaloid from *Nelumbo nucifera* leaf) in preventing stress-induced fibroblast senescence by using a hydrogen-peroxide (H_2_O_2_)-induced senescence model. We found that H_2_O_2_ treatment resulted in a significant increase in senescence-associated β-galactosidase (SA-β-gal)-positive cells. Nuciferine-treated cells, however, showed a reduction in senescent phenotype. Furthermore, we observed the key molecular markers including the senescence-associated secretory phenotype (SASP) and cell cycle regulators. The mRNA levels of CXCL1, CXCL2, IL-6, and IL-8 (pro-inflammatory cytokines) reduced significantly in nuciferine-treated cells. The extracellular IL-6 and IL-8 levels were also decreased in treated cells, whereas the key cell cycle regulators (p16 and p21) were markedly affected by nuciferine at the highest concentration. The results of the present study clearly show that the preventive activity of nuciferine against H_2_O_2_-induced senescence in dermal fibroblasts is fundamental and promising for further applications in anti-aging product research and development.

## 1. Introduction

The cellular aging process was initially identified by the limited proliferation capacity of human fibroblasts derived from embryonic tissues [1,2]. If the fibroblasts are not able to maintain proliferative potential, then they are becoming senescent. Senescent cells have a flattened shape and are high in senescence-associated β-galactosidase (SA-β-gal) activity [3,4]. They also exhibit the senescence-associated secretory phenotype or SASP with the secretion of pro-inflammatory cytokines, particularly IL-6, IL-8, and chemokines [5,6]. During aging, they undergo biochemical alterations, including a decrease in collagen gene expression and an increase in metalloproteinase I synthesis [5,6]. These changes lead to collagen fibril fragmentation and a subsequent decrement in skin density and elasticity [1]. One of the biochemical insults is an oxidant that leads to oxidative damage [7]. When human skin is exposed to numerous external stimuli, reactive oxygen species (ROS) are generated and cause fibroblast aberrant functions [1,8].

The human diploid fibroblasts offer a typical model for studying in vitro cell senescence [2]. The impact of various senescence-inducing stress signals and the strategies to circumvent them have been an important area of focus in aging skin research [9]. Several lines of evidence suggest that hydrogen peroxide (H_2_O_2_) induces fibroblast senescence and subsequent cell death [10,11]. The H_2_O_2_ is experimentally used to study oxidative-stress-induced premature senescence within a short period of time. This cellular event produces the key markers of replicative senescence cells. The H_2_O_2_-induced fibroblast senescence can be regarded as a useful model for skin aging. Furthermore, this phenomenon is preventable by some nutrients (vitamin C and E) and plant-derived phytochemicals [7,9,12,13,14].

Lotus (*Nelumbo nucifera*) is a natural food plant used as both food and herbal medicine in many Asian countries such as China, Japan, Korea, Taiwan, India, and Thailand. Nowadays, many new or unknown phytochemical compounds from *N. nucifera* leaf are still being discovered. Nuciferine belongs to the alkaloid group having various biological activities including anti-hyperlipidemia and cholesterol-lowering activity [15], and anti-inflammatory and anti-hyperuricemic effects [16]. Yan and colleagues showed that it had sedative-hypnotic and anxiolytic effects [17]. More recently, it has been reported that nuciferine exhibits protective effects against folic-acid-induced kidney injury [18], high-fat-diet-induced diabetes in C57BL/6J mice [19], hepatic steatosis in Sprague–Dawley rats [20], isoproterenol-induced myocardial infarction in Wistar rats [21], and doxorubicin-induced cardiotoxicity in vitro and in vivo [22].

Extensive studies have shown the relationship between ROS, aging, and cellular senescence. In this regard, the prevention of senescence could reduce premature aging [9]. The research work regarding nuciferine on skin aging especially using the fibroblast senescence model has been limited so far. Therefore, the main purpose of this research work was to evaluate the preventive potential of nuciferine against H_2_O_2_-induced premature senescence in dermal fibroblasts using the SA-β-gal as a biomarker. The key senescence-associated genes including CXCLl, CXCL2, IL-6, and IL-8 were also investigated. Furthermore, we performed the respective experiments in comparison with vitamin C (an antioxidant with anti-senescent activity), which was considered a positive control for the present study.

## 2. Results

### 2.1. Fibroblast Cell Viability in the Presence of Nuciferine

**Figure 1:**
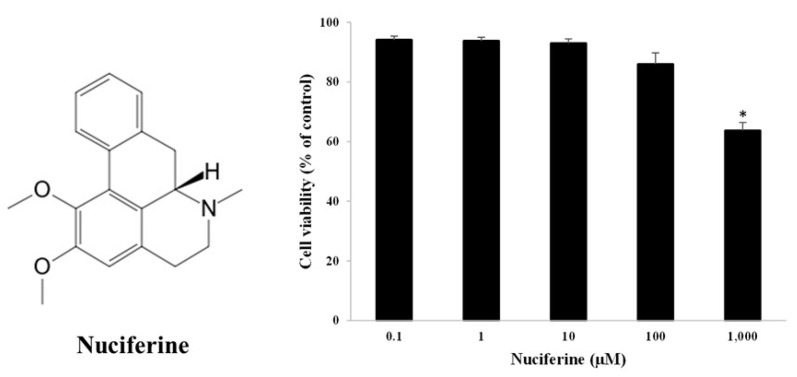
Structure of nuciferine (an alkaloid from lotus leaves). Fibroblast cell viability against nuciferine treatment (0–1000 μM) (right). Percentage of viable cells expressed as mean ± SEM from triplicate independent experiments, * indicates *p* < 0.05 of one-way ANOVA.

The dermal fibroblasts were used to assess cell viability. The cells were treated with nuciferine concentrations ranging from 0.1 to 1000 μM. After 48 h of treatment, the MTT colorimetric assay showed that the cells could survive 85–96% when exposed to 0.1–100 μM nuciferine (Figure 1). No significant cell injury was observed at less than 100 μM nuciferine when compared to control cells. However, at the highest concentration (1000 μM), nuciferine significantly reduced the cell viability down to 60%. For further study regarding cellular senescence, a nuciferine concentration less than 100 μM was preferable to implement because it was a safe and harmless concentration range.

### 2.2. Effect of Nuciferine on H_2_O_2_-Induced Senescence-Associated ß-Galactosidase Expression in Fibroblasts

**Figure 2:**
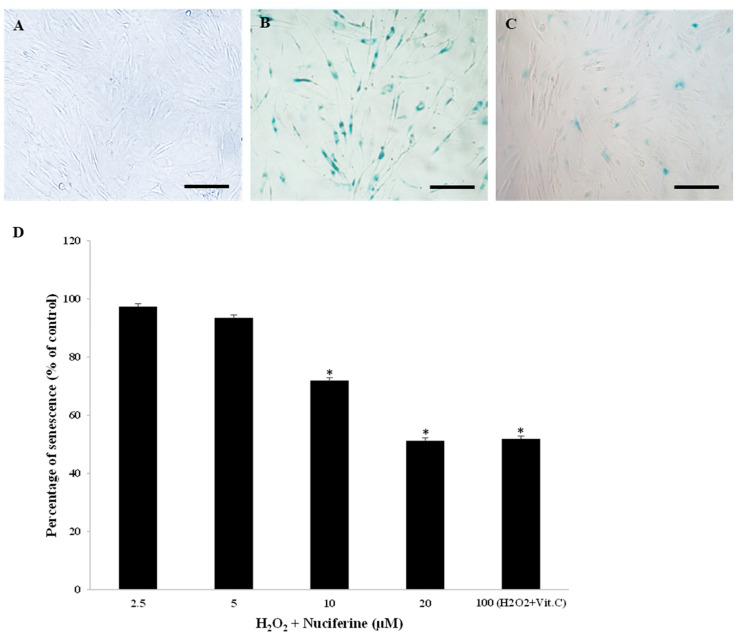
Preventive effect of nuciferine on H_2_O_2_-induced fibroblast senescence. Representative microscopic *ß*-galactosidase-positive fibroblasts (scale bar = 200 µm). (**A**): The absence of blue cells indicates fibroblasts with non-senescence activity (negative control). (**B**): Fibroblasts receiving H_2_O_2_ (300 μM) show a massive senescence activity (many blue cells) (positive control). (**C**): A small number of senescent cells can be seen in fibroblasts treated with H_2_O_2_ and nuciferine (20 μM). (**D**): The percentage of senescence was calculated after co-incubation with H_2_O_2_ and nuciferine (0–20 μM) or vitamin C (100 μM). Mean ± SEM from triplicate independent experiments, * indicates *p* < 0.05 of one-way ANOVA.

The cultured fibroblasts normally grew in a monolayer (Figure 2A). To test whether nuciferine could prevent H_2_O_2_-induced senescence-associated *ß*-galactosidase, the cells were pretreated with nuciferine prior to H_2_O_2_. Exposure to 300 µM H_2_O_2_ increased the number of senescent cells (82.35%) due to the accumulation of lysosomal *ß*-galactosidase activity from a course of replicative senescence (Figure 2B). However, this senescent appearance could be prevented by nuciferine treatment significantly (Figure 2C). Furthermore, the pre-treated cells survived the H_2_O_2_ toxicity as they could grow up to 90–100% following H_2_O_2_ exposure. The preventive effect of nuciferine was compared with ascorbic acid (a known anti-senescent compound). Figure 2D shows the preventive effect of nuciferine against H_2_O_2_, which was presented as the percentage of senescence (% of control). Nuciferine (20 μM) prevented human fibroblasts from H_2_O_2_-induced senescence significantly and equivalently to 100 μM vitamin C. Nuciferine alone at the same concentration range (2.5–20 μM) did not induce senescence. Therefore, it was safe and able to prevent fibroblast senescence. 

### 2.3. Senescence-Associated Secretory Phenotype (SASP) Gene Expression upon Nuciferine Treatment

**Figure 3:**
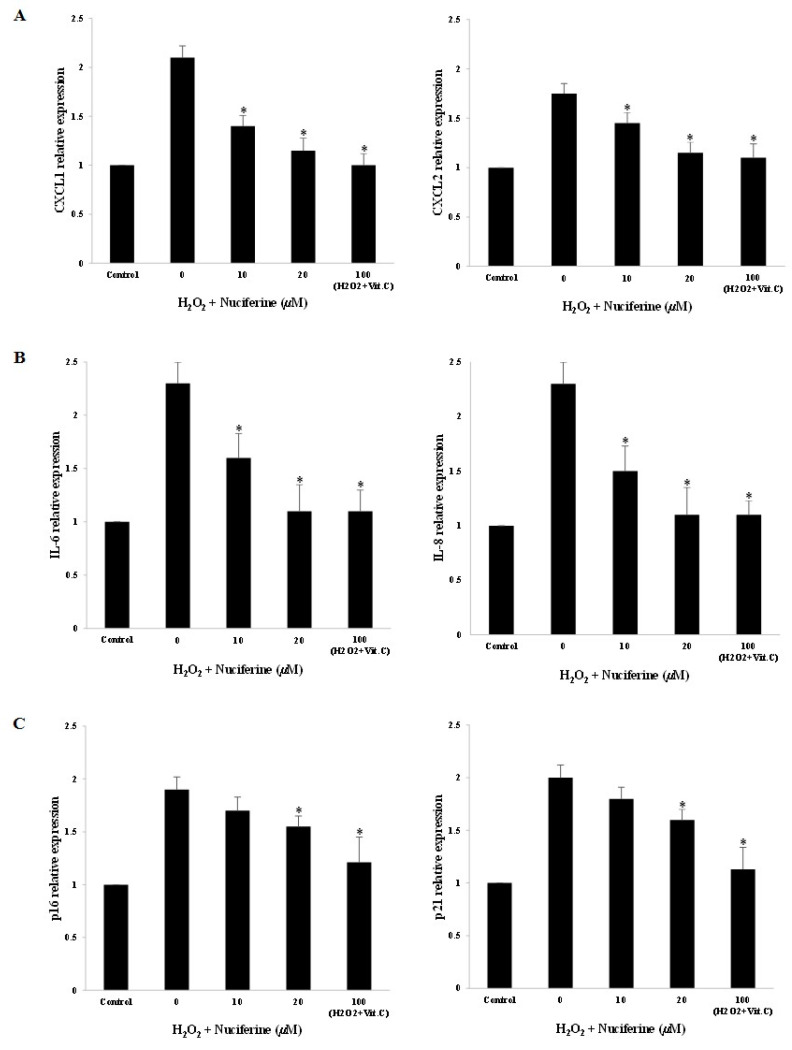
Downregulating effect of nuciferine on SASP gene expression upon H_2_O_2_-induced fibroblast senescence. After co-incubation with H_2_O_2_ (300 μM) and nuciferine (0–20 μM) or vitamin C (100 μM), the mRNA from control- and treated-fibroblasts were isolated and quantified by quantitative real-time PCR. (**A**): The expression of C-X-C motif chemokine ligands (CXCL1 and CXCL2) genes. (**B**): The interleukin (IL)-6 and IL-8 gene expression. (**C**): The expression of p16 and p21 (cell cycle inhibitors) genes. The average relative expressions of SASP genes between control- and treated cells were analyzed with one-way ANOVA (* as *p* < 0.05).

Nuciferine and vitamin C diminished the mRNA expression of senescence-associated genes including CXCL1, CXCL2, IL-6, and IL-8 in the H_2_O_2_-treated fibroblasts (Figure 3A,B). In addition, the cell-cycle inhibitors (p16 and p21) were decreased significantly (Figure 3C). Both nuciferine and vitamin C showed a similar downregulating activity on H_2_O_2_-induced fibroblast senescence. Upon nuciferine treatment, these subcellular responses could protect fibroblasts against senescent progression.

### 2.4. Reducing Effect of Nuciferine on Pro-Inflammatory Cytokine Secretion

**Figure 4:**
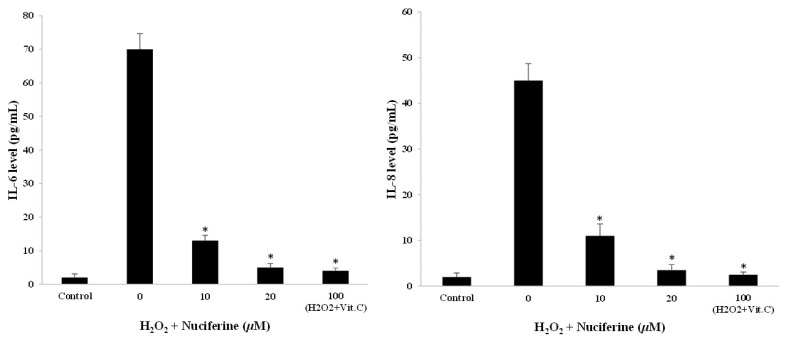
Effect of nuciferine on IL-6 and IL-8 secretion upon H_2_O_2_-induced fibroblast senescence. Cell-cultured media from non-treated and treated fibroblasts were aspirated and measured for IL-6 and IL-8 by ELISA technique. Mean ± SEM from triplicate independent experiments, * indicates *p* < 0.05 of one-way ANOVA.

The secretion of key pro-inflammatory cytokines including IL-6 and IL-8 was measured to investigate SASP occurrence. The control cells produced a small amount of both IL-6 and IL-8 levels, whereas the H_2_O_2_-treated cells released an immense amount of them. In addition, the fibroblasts receiving nuciferine had decreasing IL-6 and IL-8 gene expression levels (Figure 3B) and respective protein levels when measured by ELISA (Figure 4). Thus, the suppressive effect of nuciferine in reducing SASP could be considered an implication in senomodifying agent research.

## 3. Discussion

The utilization of plant-derived bioactive compounds in attenuating skin cell degeneration is in commercial focus. Healthy dermal fibroblasts produce a group of proteins that results in skin elasticity and flexibility. However, ROS exposure can damage the cells and lead to senescence. The early onset of cellular senescence induced by oxidative stress is termed stress-induced premature senescence [5]. Senescent fibroblast cells have a specifically enlarged cytoplasm, poor response to growth factors, and β-galactosidase activity [4,23,24]. The H_2_O_2_, when being exposed to cultured fibroblasts, induces premature senescence by imposing oxidative stress and sustained accumulation of oxidative DNA damage [10,11]. In this study, the preventive ability of nuciferine to mitigate H_2_O_2_-induced premature senescence was assessed. The results indicated that normal fibroblasts receiving H_2_O_2_ showed an increase in the percentage of SA-β-gal, while nuciferine (a lotus-derived alkaloid) could reverse it significantly, and 20 µM nuciferine treatment showed a similar preventive effect with that of vitamin C. The results corroborate with the work that demonstrated that vitamin C could inhibit aging markers in human dermal fibroblasts and mice models [11,14]. Thus, nuciferine might be considered as an active ingredient in anti-aging skincare. 

The treatment of fibroblasts with H_2_O_2_ provoked an increased expression of senescence-associated genes (Figure 3). Consequently, the respective senescence-associated secretory phenotype or SASP was also observed (Figure 4). These molecular changes are involved in senescent reduction when cells are exposed to nuciferine and could be considered one of the key senescent prevention mechanisms evoked by nuciferine. The changes were compatible with a hallmark of senescent fibroblasts [25]. A number of publications show that senescent cells have damaging effects on the tissue microenvironment [8,25]. However, the nuciferine treatment could significantly reduce the key senescence-associated genes including CXCL1, CXCL2, IL-6, and IL-8. The recent research on inflammation by Zhang et al. showed that nuciferine inhibited pro-inflammatory cytokines (TNFα and IL-6) by activating PPAR*α* and PPAR*γ* in lipopolysaccharide (LPS)-induced RAW 264.7 macrophage cells [26]. In 2017, the inflammation research by Wu and colleagues demonstrated that the molecular mechanism of nuciferine on LPS-treated macrophage cells was related to the downregulation of toll-like receptor-4 expression and nuclear factor (NF)-*κ*B activation [27]. Moreover, Wen and colleagues showed that nuciferine also protected IL-1β-induced rat chondrocyte inflammation by suppressing the activation of NF-*κ*B and the PI3K/Akt pathway [28]. The previous seminal studies plausibly explain the mechanistic basis for senescent prevention of nuciferine in the present work. Furthermore, according to recent research on plant extract and inflammation, it has been demonstrated that natural compounds with antioxidant activities can prevent senescent progression by modulating SASP factors and associated genes [10,14,29]. Based on the obtained results and previous work, nuciferine could reduce inflammatory mediators and prevent fibroblasts from H_2_O_2_ toxicity. 

The cell cycle regulators are some of the markers of cellular senescence. The previously published reports demonstrated that the dermal fibroblasts receiving H_2_O_2_ underwent G0/G1 arrest. This cellular adaptation was also observed concomitantly with ROS accumulation and oxidation-associated damage [10,11]. In this regard, oxidative stress could result in DNA damage and cell cycle arrest [6]. The two cyclin-dependent kinase inhibitors (CDKIs), p16 and p21, collaborate to maintain the dephosphorylated form of RB protein, thereby contributing to strong irreversible cell cycle arrest. Consistently, the high expression of p16 and p21 is often used as senescence markers [30]. Nuciferine (20 µM) lowered both p16 and p21 gene expression significantly. Research by Kang et al. demonstrated that nuciferine slightly increased the sub-G1 peak in cancer cells but induced G1 arrest in MDA-MB-231 cells and G2 arrest in MCF-7 cells [31]. Another issue is that the investigation of the role of serotonin (5-hydroxytryptamine, 5-HT) and the 5-HT1A receptor has been established in the regeneration of tissues, such as the liver and spinal cord. It was found in the knock-out cell model that the fibroblasts lacking the 5-HT1A receptor did not alter DNA synthesis and cell cycle substantially, and the fibroblast viability was also not affected [32]. In 2018, nuciferine had been evaluated for its antagonist potency and selectivity against 5-HT2 receptor subtypes (5-HT2A, 5-HT2B, and 5-HT2C) [33]. However, it could agonize the 5-HT7 and 5HT1A receptor subtypes partially. Therefore, nuciferine had both antagonistic and agonistic activities, which varied between subtypes. Recently, natural polyphenols with antioxidant capacity, including mangiferin, apigenin, and kaempferol, were able to prevent senescence by downregulating SASP-associated gene expression in senescent fibroblasts [12,14]. Thus, nuciferine with anti-inflammatory activity was the promising natural compound in preventing H_2_O_2_-induced fibroblast senescence.

The secretory phenotype is another hallmark of H_2_O_2_-induced senescence. Fibroblasts treated with H_2_O_2_ produce the key pro-inflammatory cytokines, particularly IL-6, IL-8, and chemokines, thus typically serving as senescent markers [6]. Our experiment using ELISA to measure both IL-6 and IL-8 levels indicated that nuciferine not only downregulated the gene expression but also reduced the production and secretion. The present study is in accordance with the previous seminal work. Some investigations regarding naturally occurring flavonoids, such as apigenin, kaempferol, and vitamin C, on H_2_O_2_-triggered fibroblast senescence have demonstrated the cytoprotective potential against the production of SASP cytokines (IL-6 and IL-8) as well as gene expression [7,11,13]. In line with the previous seminal work, the decrease in pro-inflammatory cytokines upon nuciferine treatment emphasized the anti-senescent potential in dermal fibroblasts, making it suitable for further skin anti-aging product research.

## 4. Conclusions

The present work showed that nuciferine could mitigate H_2_O_2_-mediated premature senescence. It might act as a potential ingredient with antioxidant and anti-inflammation and could be used in place of vitamin C in anti-aging product research and development.

## 5. Materials and Methods

### 5.1. Chemicals

All chemicals and solvents were analytical grade. The H_2_O_2_ was purchased from Merck Co. (Darmstadt, Germany). 3-[4,5-dimethylthiazol-2-yl]-2,5 diphenyl tetrazolium bromide (MTT) and nuciferine were purchased from Sigma Chemical Co. (St. Louis, MO, USA). The Eagle’s minimum essential medium (EMEM), fetal bovine serum (FBS), and antibiotic-antimycotic reagent for cell culture were purchased from Thermo Fisher Scientific Inc. (Waltham, MA, USA). The qPCR reagents were from Takara Inc. (Kyoto, Japan). Pro-inflammatory cytokines were quantified using ELISA kits from Abcam (Waltham, MA, USA). Primers were purchased from Macrogen (Seoul, Korea).

### 5.2. Cell Culture

The human dermal fibroblast (BJ, CRL-2522™) was from ATCC. Cells were cultured in EMEM containing 2 mM L-glutamine, 1% non-essential amino acids, 10% FBS, 1 mM sodium pyruvate, and 1500 mg/L of sodium bicarbonate until reaching 80% confluency for the in vitro assays. Fibroblasts were seeded 1 × 10^3^ cells/well for a 96-well plate for testing the cytotoxic effect of nuciferine. For fibroblast senescent evaluation, 1 × 10^5^ cells were inoculated into each well in 6-well plates [12].

### 5.3. Cell Viability Assessment by MTT Colorimetric Assay

The mitochondrial esterase activity of fibroblasts was considered an indicator of cell viability. Following the incubation of various concentrations of nuciferine (0–1000 μM) for 48 h, the fibroblasts were washed twice with an incomplete medium and then exposed to 20 µL of MTT dye for 4 h. The formazan crystal formed regarding cell viability was dissolved in DMSO and measured at 540/630 nm (Biotek Cytation 5 multi-mode reader, Santa Clara, CA, USA) for further calculation of fibroblast proliferation [34].

### 5.4. Fibroblast Senescence Induction with H_2_O_2_

The fibroblasts were cultured in EMEM under an atmosphere of 5% CO_2_ at 37 °C. The fibroblasts were pre-treated with various concentrations of nuciferine (0–20 μM) for 24 h. For the cellular senescence induction, the cells were exposed to 300 μM H_2_O_2_ for 4 h and then treated with or without nuciferine, for an additional 24 h. We also compared the tested groups with a positive control group by incubating the cells with 100 μM ascorbic acid [11].

### 5.5. Cellular Senescence Assay

The SA-β-gal was indicative of fibroblast senescence. Following Maier’s protocol with modification [4], the culture media were aspirated after 24 h treatment with H_2_O_2_ and nuciferine, and the cells were washed three times with 1 mL of PBS. Then, the cells were fixed with 250 µL of 4% *p*-formaldehyde for 5 min at room temperature. The cells were washed 3 times with gentle shaking in 1 mL of PBS. After the last wash, the SA-β-gal staining solution (pH 6.0, 250 µL) was added to each well and incubated in a dark place at 37 °C overnight. The solution contained 1 mg/mL of X-galactoside, 40 mM citric acid, 5 mM potassium ferrocyanide, 5 mM potassium ferricyanide, 150 mM sodium chloride, and 2 mM magnesium chloride. By applying Maier’s protocol, the proportion of cells positive for SA-β-gal activity could be easily determined by counting the number of blue cells in the total population. Senescent cells (positive staining) were quantified based on the presence of blue-stained cells. Cell populations in random fields were counted under an inverted microscope (Olympus CKX53, Tokyo, Japan). To indicate the preventive effect of nuciferine against H_2_O_2_ treatment, the data were expressed as the percentage of senescence (% of control).

### 5.6. RNA Isolation

The RNA from non-treated and treated cells was extracted and isolated using TRIzol™ reagent (Ambion^®^, Invitrogen, Waltham, MA, USA). The reagent was added to the cells to isolate separate fractions of RNA, DNA, and proteins. Chloroform was then added to the homogenate and allowed to separate into a clear upper aqueous layer (RNA-containing fraction). RNA was precipitated from the aqueous layer with isopropanol. Isolated RNA was resuspended in ultrapure water and kept at −70 °C for complementary DNA synthesis.

### 5.7. Quantitative Real-Time PCR

Total RNA was reverse-transcribed into cDNA using the PrimeScriptTM RT reagents kit (Takara Bio Inc., Kyoto, Japan). Real-time quantitative PCR reactions were performed using the SYBR^®^ Premix Ex Taq™ kit (Takara Bio Inc., Kyoto, Japan) in the CFX Duet Real-Time PCR System (Bio-rad Laboratories, Inc., Hercules, CA, USA). The primers used in this research are shown in Table 1. The expression of each gene was normalized to β-actin and was presented as a relative expression ratio (2^−ΔΔCt^, ΔΔCt = ΔCt_target_ − ΔCt*_β_*_-actin_).

### 5.8. Enzyme-Linked Immunostaining Assay for Interleukin Measurement

The assay was according to the company’s protocol (Abcam, Waltham, MA, USA). In brief, the culture media were centrifuged at 2000× *g* for 10 min, and then the supernatants were diluted 1:500 with the sample diluent. The standard curve was plotted using IL-6 and IL-8 concentrations ranging from 0 to 250 pg/mL. For the assay, the standard IL-6/IL-8 and samples (50 µL) were added to the microplate and then followed by 50 µL of the antibody cocktail. After 1 hr of incubation, 100 µL of 3,3′,5,5′-tetramethylbenzidine or TMB (colorimetric dye) was added to develop the specific color resulting from oxidation of TMB and was measured at 450 nm (Biotek Cytation 5 multi-mode reader, Santa Clara, CA, USA). 

### 5.9. Statistical Analysis

Data were expressed as the mean ± standard error of means (SEM) of triplicate independent experiments. All treated groups were compared by one-way analysis of variance (ANOVA), followed by Tukey’s test. Differences were considered statistically significant at *p* < 0.05.

## Figures and Tables

**Table 1:** Primer sequences for qPCR.

Target Genes	Forward Primer	Reverse Primer
CXCL1	AGCTTGCCTCAATCCTGCATCC	TCCTTCAGGAACAGCCACCAGT
CXCL2	GGCAGAAAGCTTGTCTCAACCC	CTCCTTCAGGAACAGCCACCAA
IL-6	GACTGTGCACTTGCTGGTGGAT	ACTTCCTCACCAAGAGCACAGC
IL-8	GAGAGTGATTGAGAGTGGACCAC	CACAACCCTCTGCACCCAGTTT
p16	CTCGTGCTGATGCTACTGAGGA	GGTCGGCGCAGTTGGGCTCC
p21	AGGTGGACCTGGAGACTCTCAG	CGGTGTCTGTAGTGGCTTGACT
β-actin	CACCATTGGCAATGAGCGGTTC	AGGTCTTTGCGGATGTCCACGT

## Data Availability

Not applicable.

## Sample Availability

Samples of the compounds are not available from the authors.
